# A complete sequence of mitochondrial genome of *Neolamarckia cadamba* and its use for systematic analysis

**DOI:** 10.1038/s41598-021-01040-9

**Published:** 2021-11-02

**Authors:** Xi Wang, Ling-Ling Li, Yu Xiao, Xiao-Yang Chen, Jie-Hu Chen, Xin-Sheng Hu

**Affiliations:** 1 College of Forestry and Landscape Architecture, South China Agricultural University, Guangdong, 510642 China; 2 Guangdong Key Laboratory for Innovative Development and Utilization of Forest Plant Germplasm, South China Agricultural University, Guangdong, 510642 China; 3 Science Corporation of Gene (SCGene), Guangzhou, 510000 China

**Keywords:** Next-generation sequencing, Phylogenetics

## Abstract

*Neolamarckia cadamba* is an important tropical and subtropical tree for timber industry in southern China and is also a medicinal plant because of the secondary product cadambine. *N. cadamba* belongs to Rubiaceae family and its taxonomic relationships with other species are not fully evaluated based on genome sequences. Here, we report the complete sequences of mitochondrial genome of *N. cadamba*, which is 414,980 bp in length and successfully assembled in two genome circles (109,836 bp and 305,144 bp). The mtDNA harbors 83 genes in total, including 40 protein-coding genes (PCGs), 31 transfer RNA genes, 6 ribosomal RNA genes, and 6 other genes. The base composition of the whole genome is estimated as 27.26% for base A, 22.63% for C, 22.53% for G, and 27.56% for T, with the A + T content of 54.82% (54.45% in the small circle and 54.79% in the large circle). Repetitive sequences account for ~ 0.14% of the whole genome. A maximum likelihood (ML) tree based on DNA sequences of 24 PCGs supports that *N. cadamba* belongs to order Gentianales. A ML tree based on *rps*3 gene of 60 species in family Rubiaceae shows that *N. cadamba* is more related to *Cephalanthus accidentalis* and *Hymenodictyon parvifolium* and belongs to the Cinchonoideae subfamily. The result indicates that *N. cadamba* is genetically distant from the species and genera of Rubiaceae in systematic position. As the first sequence of mitochondrial genome of *N. cadamba*, it will provide a useful resource to investigate genetic variation and develop molecular markers for genetic breeding in the future.

## Introduction

*Neolamarckia cadamba* is one of two species (*N. macrophylla* for the other species) in genus *Neolamarckia* of Rubiaceae^1^, one of the largest families in flowering plants. The species is naturally distributed in Vietnam, Malaysia, Myanmar, India and Sri Lanka, and mainly grows in Guangdong, Guangxi and Yunnan Provinces in China. It grows in the habitat of high temperature and humidity, with the average temperature of 20–24 °C and the annual precipitation of 1200–2400 mm, and also in the fertile, loose and humid soil or in the humid sandy soil. *N. cadamba*, aka a miraculous tree, is a fast-growing species^2^ and commercially important materials. Its wood is good for building construction, wood board making, furniture, pulp and paper production^3^. In addition, the tree fruits can be used for nutraceutical enriched beverage^4^. Leaves are used as woody forage to feed livestock^5^ and have effects of antibacterial and anti-inflammatory to animals^6^. One particular value is that the species has enormous pharmacological implications due to its rich secondary metabolites (e.g., phenols and alkaloids)^6–9^. The monoterpenoids, alkaloids and triterpenoids are potentially used for medicinal purposes^10,11^. *N. cadamba* is exploited for antimicrobial, wound healing and antioxidant activities^12–14^ and for traditionally curing a number of diseases, such as diabetes, anaemia and infectious diseases^6^. The species as a medicinal plant is appreciated in South Asia^15,16^ and shows enormous medical implications.

Although *N. cadamba* is a miraculous tree, the absence of reference genome limits the molecular and evolutionary studies of this species. Current genetic studies of this species cover broad areas, including provenance trials^17,18^, propagation through tissue culture^19,20^, transcriptome analysis of gene expressions^21,22^, single nucleotide polymorphisms (SNPs) and SNP-trait association^23,24^, expressed sequence tags (ESTs) of xylem tissues^25^ and gene discovery in the developing xylem tissue^26^. Nevertheless, few studies with molecular markers have been reported on population genetic structure, phylogeography and molecular systematics^27^. This necessitates determination of the genomic sequences to understand the genetic basis of these characters (rapid growth, quality timber, secondary metabolites, etc.), to develop appropriate molecular markers for breeding program, and to gain insights into the evolutionary history of this species.

To develop markers for population genetics and phylogenetic analysis, we here sequenced and reported the mitochondrial genome of this species. The well-known features of mitochondrial DNA (mtDNA) in plants include (i) maternal inheritance in angiosperms, (ii) haplotype per cell, (iii) intra-molecular recombination between repeats^28^, and (iv) the number of females as its population size (*N*_*f*_). These features differ from those of nuclear genomes, which correspondingly exhibits (i) biparental inheritance, (ii) diploid per cell, (iii) inter-chromosome recombination and relatively high mutation rates, and (iv) large effective population sizes (2*N*_*e*_) of nuclear genes ($2N_{e} = 4N_{f}$ under 1:1 sexual ratio)^29^. Compared with chloroplast and nuclear DNAs, mtDNA has generally a lower mutation rate in plants^30^. Thus, mtDNA sequences are useful for studying the long-term phylogenetic relationships at the level of species or higher order, and also for studying other perspectives of evolutionary relationships, such as lineage sorting, hybridization and cytonuclear interactions^31^.

Although three major subfamilies in Rubiaceae are delineated, including Rubioideae^32^, Ixoroideae^33^ and Cinchonoideae^34^, systematic position of *N. cadamba* remains to be evident. From the morphological characters, *N. cadamba* is classified into subfamily Cinchonoideae, tribe Naucleeae^1^. Based on the cytogenetic study^35,36^, *N. cadamba* has 44 chromosomes (2n) and belongs to subfamily Cinchonoideae, tribe Naucleeae and Subtribes Neolamarckinae^37^. In this study, we determined the complete mtDNA sequence of *N. cadamba* and detailed its characteristics. Based on the mtDNA sequence, we then evaluated the phylogenetic relationships among families and genera of Rubiaceae to gain insights into the taxonomic position of *N. cadamba*.

## Results and discussion

### Assembly of mitochondrial genome

**Figure 1:**
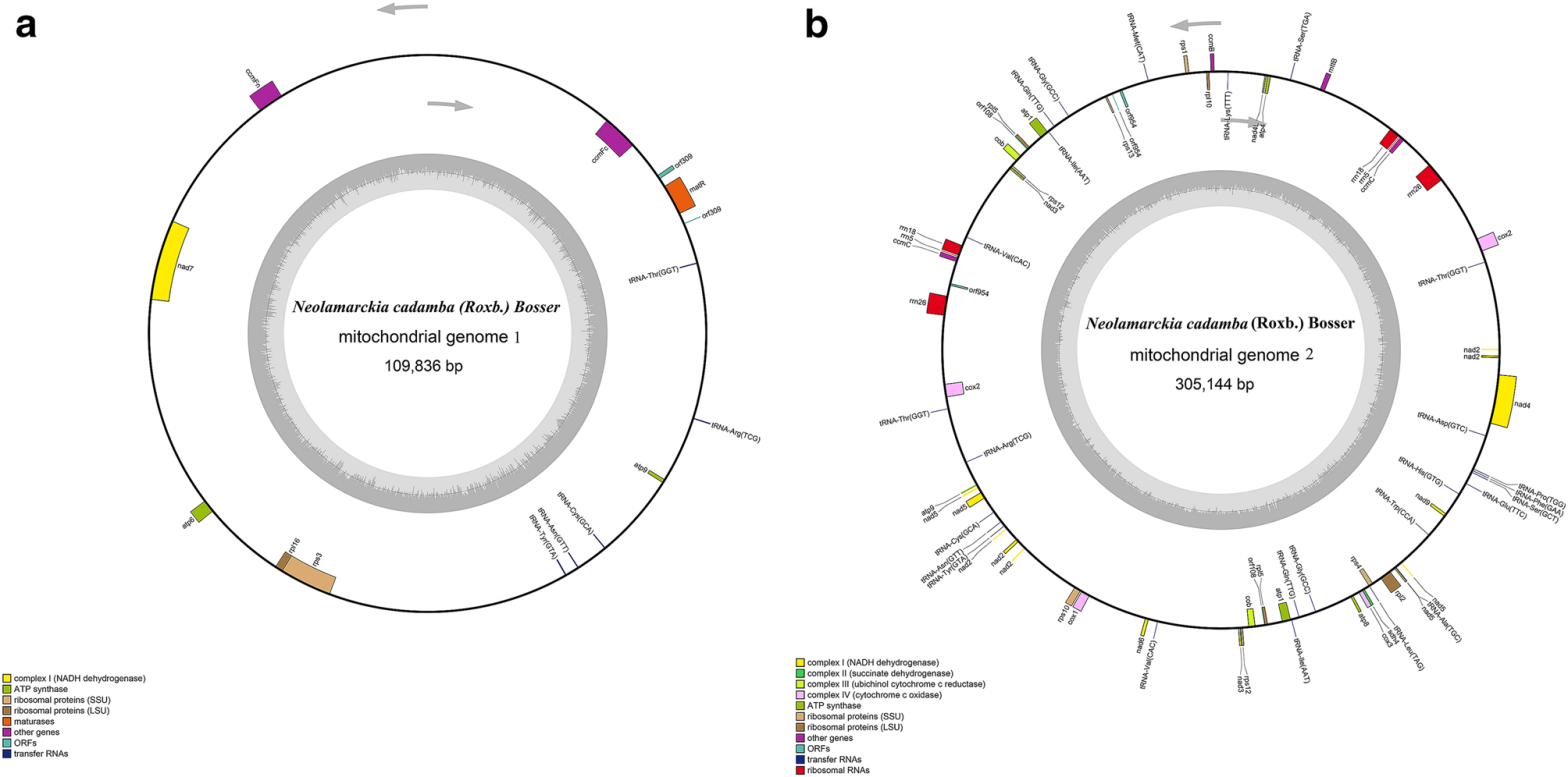
Two circular maps of the mitochondrial genome of *Neolamarckia cadamba.*

MtDNA sequence of *N. cadamba* was determined using PacBio sequencing technique and was successfully assembled in two genome circles. This probably reflects the feature of rapid evolution of structure of plant mitochondrial genomes^38–40^. Figure 1 shows two parts of circular structure of the mitochondrial genome, designated as genomes 1 and 2. The genome 1 has 109,836 bp (GenBank Access No. MT320890). It contains 14 genes (Table 1), including 7 protein-coding genes (PCGs), 5 transfer RNA genes, and 2 other genes *(ccm*Fc, *ccm*Fn). The PCGs are 1 NADH dehydrogenase genes (*nad*7), 2 ATP synthase genes (*atp*6, *atp*9), 2 ribosomal proteins genes (*rpl*18, *rps*3), 1 maturases gene, and 1 ORF. Four PCGs (*nad*7, *rpl1*6, *rps*3, *atp*9), 4 tRNA genes, and 1 other gene (*ccm*Fc) are on the N-strand, and genes of 1 PCG *atp*6, 1 tRNA gene and 1 other gene (*ccm*Fn) are on the J-strand. There is only one overlapping region (110 bp in length) between *rpl*16 and *rps*3 in genome 1.

**Table 1:** Annotations and characteristics of mitochondrial genome 1 of *Neolamarckia cadamba.*

Gene	Strand*	Position	Length (bp)	GC content (%)	Initiation codon	Termination codon	Intergenic nucleotides (bp)
*tRNA-Thr*	N	4421–4492	72	51.39			
*orf*309	J	7042–7092	309	0.45	ATG	TAA	2549
		10,274–10,531					
*matR*	J	7756–9723	1968	51.78	ATG	TAG	663
*ccmFc*	N	12,975–13,523	1317	0.45	ATG	TGA	3251
		14,475–15,242					
*ccmFn*	J	37,088–38,716	1629	45.30	ATG	TAA	21,845
*nad*7	N	47,657–47,917	1185	0.44	ATG	TAG	8940
		49,703–50,413					
		51,678–51,746					
		52,647–52,790					
*atp*6	J	66,186–67,004	819	37.61	ACG	TAA	13,395
*rpl*16	N	72,277–72,792	516	44.57	ATG	TAA	5272
*rps*3	N	72,683–74,287	1674	0.42	ATG	TAG	−110
		76,033–76,101					
*tRNA-Tyr*	N	91,413–91,496	84	50.00			15,311
*tRNA-Asn*	N	92,321–92,392	72	52.78			824
*tRNA-Cys*	N	94,407–94,478	72	51.39			2014
*atp*9	N	99,941–100,186	246	45.53	ATG	TAA	5462
*tRNA-Arg*	J	104,397–104,467	71	43.66			4210

*J stands for the direction of a gene from 5′ to 3′, and N for the direction of a gene from 3′ to 5′.

The mitochondrial genome 2 is 305,144 bp in length (GenBank Access No. MT364442). The genome 2 contains 69 genes (Table 2), including 33 PCGs, 26 transfer RNA genes, 6 ribosomal RNA genes, and 4 other genes (*ccm*C*, mtt*B*, ccm*B*, ccm*C). The PCGs are 8 NADH dehydrogenase genes (*nad*4L*, nad*2*, nad*3*, nad*5*, nad*6*, nad*3*, nad*9*, nad*4), 1 succinate dehydrogenase genes (*sdh*4), 2 ubichinol cytochrome reductase genes, 4 cytochrome c oxidase genes, 5 ATP synthase genes (*atp*4*, atp*1*, atp*9*, atp*1*, atp*8), 10 ribosomal proteins genes (6 *rps*, 4 *rpl*), and 3 ORFs. The 15 PCGs (*nad*4L*, nad*2*, nad*3*, nad*9*, atp*4*, atp*1*, cob, cox*2*, rpl*10*, rpl*5*, rps*13*, rps*12*, rps*4*, orf*954*, orf*108), 10 tRNA genes, and 3 rRNA genes and 1 other gene (*ccm*C) are located on the N-strand, and the remaining 18 PCG genes, 16 tRNA genes, 3 rRNA genes and 3 other genes (*mtt*B*, ccmB, ccm*C) are on the J-strand. There are two overlapping regions, with 73 bp overlapping between *cox*3 and *sdh*4 and 817 bp overlapping between *rps*4 and *tRNA-Leu*. There are certain intergenic sequences among adjacent genes in the remaining genes, indicating relatively low density of gene distribution along the genome. This is consistent with the patterns of other plants where non-coding regions are the important parts in consisting of mitochondrial genome^40–42^.

**Table 2:** Annotations and characteristics of mitochondrial genome 2 of *Neolamarckia cadamba.*

Gene	Strand*	Position	Length (bp)	GC content (%)	Initiation codon	Termination codon	Intergenic nucleotides (bp)
*nad2*	N	1–153	1467	38.72	ATG	TAA	
		186,243–186,401					
		188,825–189,397					
		190,869–191,057					
		303,715–304,107					
*tRNA-Thr*	N	15,576–15,647	72	51.39			15,422
*cox2*	J	17,577–17,996	783	40.74	ATG	TAA	1929
		19,517–19,879					
*rrn26*	N	31,879–35,307	3429	50.22			11,999
*ccm*C	N	41,488–42,240	753	43.69	ATG	TAA	6180
*rrn*5	N	42,414–42,529	116	53.45			173
*rrn*18	N	42,691–44,531	1841	53.56			161
*mtt*B	J	57,829–58,668	840	41.79	ATG	TAG	13,297
*tRNA-Ser*	J	64,038–64,125	88	51.14			5369
*atp*4	N	67,536–68,123	588	42.01	ATG	TGA	3410
*nad*4L	N	68,311–68,613	303	35.97	ATG	TAA	187
*tRNA-Lys*	N	75,008–75,082	75	46.67			6394
*ccm*B	J	77,440–78,060	621	41.38	ATG	TGA	2357
*rpl*10	N	78,351–78,839	489	41.51	ATG	TAA	290
*rps*1	J	81,832–82,437	606	42.08	ATG	TAA	2992
*tRNA-Met*	J	89,045–89,121	77	44.16			6607
*orf*954	N	93,928–94,413	954	43.50	ATG	TAA	4806
		95,766–95,846					
		140,865–141,251					
*rps*13	N	96,776–97,126	351	39.32	ATG	TGA	929
*tRNA-Gly*	J	104,293–104,366	74	54.05			7166
*tRNA-Gln*	J	107,333–107,404	72	47.22			2966
*tRNA-Ile*	N	108,714–108,789	76	35.53			1309
*atp*1	J	108,877–110,406	1530	43.86	ATG	TAA	87
*rpl*5	J	112,970–113,524	555	42.16	ATG	TAA	2563
*orf*108	J	113,526–113,633	108	35.19	ATG	TAG	1
*cob*	J	115,168–116,349	1182	40.52	ATG	TGA	1534
*rps*12	N	117,371–117,748	378	43.65	ATG	TGA	1021
*nad*3	N	117,797–118,153	357	40.62	ATG	TAA	48
*tRNA-Val*	N	132,275–132,334	60	50.00			14,121
*rrn*18	J	133,925–135,765	1841	53.56			1590
*rrn*5	J	135,927–136,041	115	53.04			161
*ccm*C	J	136,215–136,967	753	43.69	ATG	GAA	173
*rrn*26	J	143,147–146,575	3429	50.22			6179
*cox*2	N	158,575–158,937	783	40.74	ATG	TAA	11,999
		160,458–160,877					
*tRNA-Thr*	J	162,807–162,878	72	51.39			1929
*tRNA-Arg*	N	172,669–172,739	71	43.66			9790
*atp*9	J	176,950–177,195	246	45.53	ATG	TAA	4210
*nad*5	J	177,495–177,722	1986	40.89	ATG	TAA	299
		178,573–179,787					
		261,418–261,810					
		262,913–263,062					
*tRNA-Cys*	J	182,658–182,729	72	51.39			2870
*tRNA-Asn*	J	184,744–184,815	72	52.78			2014
*tRNA-Tyr*	J	185,640–185,723	84	50.00			824
*rps*10	J	202,042–202,293	333	37.84	ACG	TGA	16,318
		203,144–203,224					
*cox*1	J	203,424–205,007	1584	42.87	ATG	TAA	199
*nad*6	J	215,496–216,113	618	40.13	ATG	TAA	10,488
*tRNA-Val*	J	217,699–217,758	60	50.00			1585
*nad*3	J	231,877–232,233	357	40.62	ATG	TAA	14,118
*rps*12	J	232,282–232,659	378	43.65	ATG	TGA	48
*cob*	N	233,681–234,862	1182	40.52	ATG	TGA	1021
*orf*108	N	236,398–236,505	108	35.19	ATG	TAG	1535
*rpl*5	N	236,507–237,061	555	42.16	ATG	TAA	1
*atp*1	N	239,625–241,154	1530	43.86	ATG	TAA	2563
*tRNA-Ile*	J	241,242–241,317	76	35.53			87
*tRNA-Gln*	N	242,627–242,698	72	47.22			1309
*tRNA-Gly*	N	245,665–245,738	74	54.05			2966
*atp*8	J	252,332–252,811	480	41.04	ATG	TAA	6593
*cox*3	J	253,922–254,719	798	43.23	ATG	TGA	1110
*sdh*4	J	254,647–255,078	432	40.74	ATG	TGA	−73
*rps*4	N	256,045–256,875	831	39.47	ATG	TAA	966
*tRNA-Leu*	J	256,059–256,123	65	46.15			−817
*rpl*2	J	258,510–259,376	978	49.49	ATG	TAA	2386
		260,429–260,479					
		260,484–260,543					
*tRNA-Ala*	J	261,648–261,712	65	40.00			1104
*tRNA-Trp*	N	269,778–269,851	74	52.70			8065
*nad*9	N	274,043–274,615	573	41.19	ATG	TAA	4191
*tRNA-His*	N	278,687–278,761	75	60.00			4071
*tRNA-Glu*	J	280,888–280,959	72	50.00			2126
*tRNA-Ser*	J	283,072–283,159	88	44.32			2112
*tRNA-Phe*	J	283,411–283,484	74	47.30			251
*tRNA-Pro*	J	283,740–283,814	75	54.67			255
*tRNA-Asp*	N	289,908–289,981	74	63.51			6093
*nad*4	J	292,242–292,703	1488	40.59	ATG	TGA	2260
		294,113–294,628					
		297,800–298,219					
		300,710–300,799					

*J stands for the direction of a gene from 5′ to 3′, and N for the direction of a gene from 3′ to 5′.

### Characteristics of nucleotide composition

The two genome circles slightly differ in nucleotide composition (SI Table 1). Genome 1 has a high content of the T base but a low content of the G base. The AT content is 54.45% and the four types of bases are 29,521 bp of A (26.88%), 30,287 bp of T (27.57%), 25,616 bp (23.32%), and 24,412 bp of G (22.23%). Genome 2 has a high content of the T base but a low content of the C base. The AT content is 54.94%, and the four bases are 83,584 bp of A (27.39%), 84,075 bp of T (27.55%), 68,286 bp of C (22.38%), and 69,089 bp of G (22.64%). The AT content is slightly higher than the GC content. The relatively high AT content was also reported in other plant species^43^ or animal species^44^.

Besides the AT or GC content, the AT-and GC-skews are often used to assess the nucleotide-compositional differences in mitochondrial genomes^45^. From SI Table 1, both AT- and GC-skews in genome 1 are negative (AT-skew = −0.0128 and GC-skew = −0.0241), indicating that genome 1 has a higher percentage of T and C than A and G, respectively. Both AT- and GC-skews are negative in PCG sequences (AT-skew = −0.0408 and GC-skew = −0.0501). However, the AT-skew in tRNAs is positive (0.0430), indicating that these genes have a higher percentage of A than T. The GC-skew in tRNAs is negative (−0.1135), indicating that these genes have a higher percentage of G than C.

In genome 2, the AT-skew (−0.0029) is negative but the GC-skew (0.0058) is positive (SI Table 1), indicating that genome 2 has a higher percentage of T and G than A and C, respectively. The GC-skews in both PCGs (−0.0115) and rRNAs (−0.1242) are negative, but positive in tRNAs (0.0449). The AT-skews are negative in PCGs (−0.0569), tRNA (−0.0289) and rRNAs (−0.0864). The extents of both AT- and GC-skews are greater in rRNAs than in PCGs and tRNAs. Generally, the extents of AT-and GC-skews in both genomes 1 and 2 are small, comparable to the pattern in mitochondrial genomes of *Pyrus pyrifolia* (AT-skew = 0.004, GC-skew = 0)^46^ but different from that of animal species *Ledra auditura* ^44^ (AT-skew = 0.22 and GC-skew = 0.12).

### Protein-coding genes and codon usage

Codon usage bias is an important character of a genome since it is associated with gene expression^47,48^, the base composition of genes^49^, amino acid composition^50^, GC content^51^, the length of a gene^52^ and tRNA richness^53,54^. Large differences in the codon usage of genes often occur among different species and organisms^52^.

The mitochondrial genome of *N. cadamba* harbors a total of 83 coding genes and 45,639 bp in length, accounting for about 11% of the entire mitochondrial genome. This density is greater than those of watermelon (*Citrullus lanatus*; 10.3% of 379,236 bp), zucchini (*Cucurbita pepo*; 3.9% of 982,833 bp)^55^ and neem (*Azadirachta indica* A. Juss; 7.7% of 266,430 bp)^56^ mitochondrial genomes. The base composition of the whole mtDNA of *N. cadamba* is 27.26% for A, 22.63% for C, 22.53% for G and 27.56% for T, exhibiting a AT-biased pattern, with the A + T content of 54.82%. The AT-biased pattern is frequently observed in both plant and animal mitochondrial genomes^57^.

**Figure 2:**
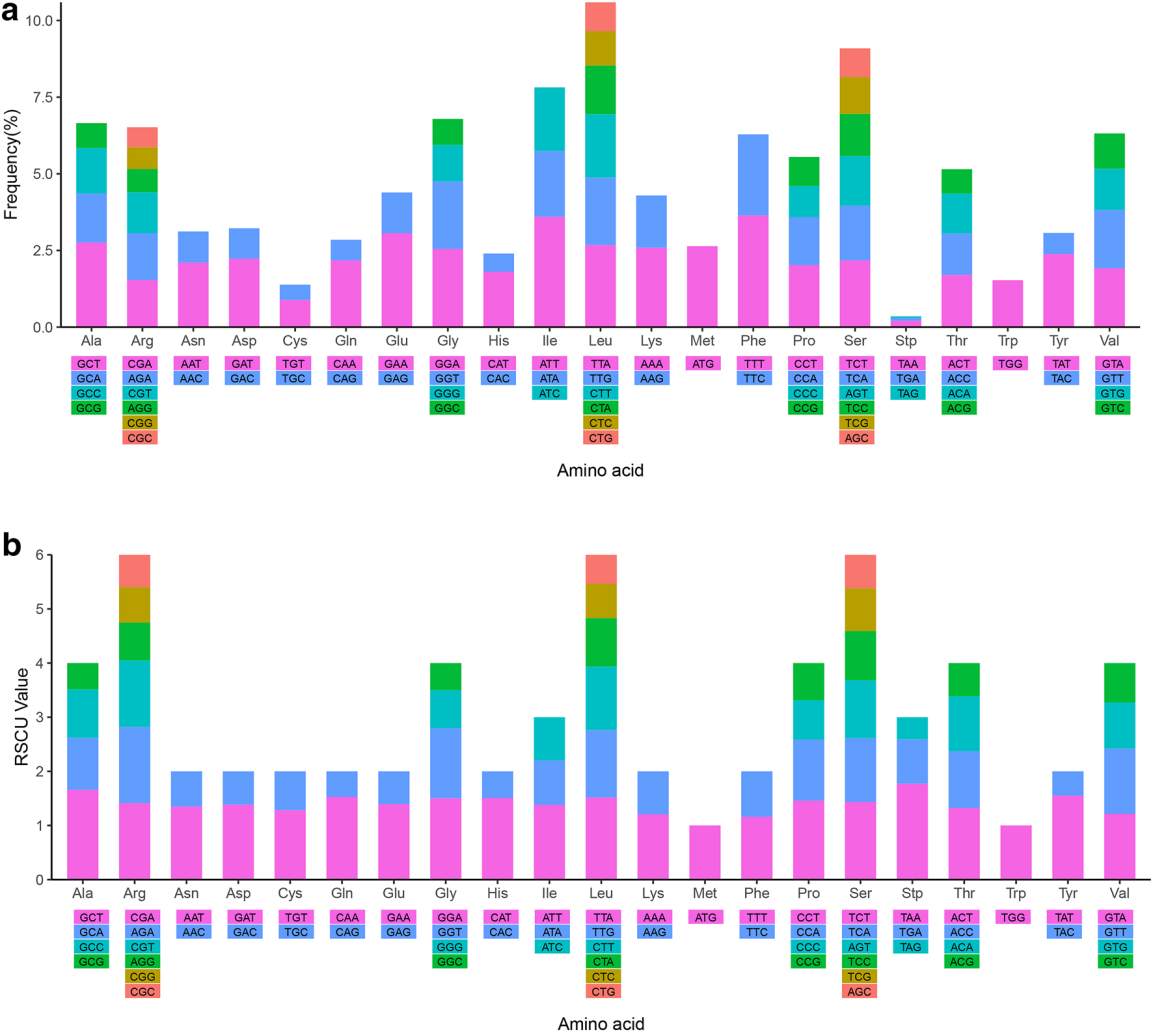
Amino acid frequency and RSCU value of protein-coding genes in mitochondrial genome of *Neolamarckia cadamba*.

The mitochondrial genomic protein-coding genes of *N. cadamba* are 37,521 bp in length, accounting for 83.03% of all coding genes. The 40 protein-coding genes encode a total of 12,507 codons. Figure 2 shows the frequencies of different amino acids in the protein-coding genes where the amino acid Leu is most frequently used, followed by Ser, Ile and Gly. From the values of relative synonymous codon usage (RSCU), there are 32 optimal codons (RSCU > 1): TAA, GCT, TAT, CAA, CAT, GGA, TTA, TCT, CCT, AGA, CGA, GAA, GAT, ACT, AAT, ATT, GGT, TGT, GTT, CTT, GTA, CGT, TTG, TCA, AAA, TTT, CCA, AGT, ACC, GCA, ATG, and TGG. The remaining 32 codons are non-optimal (RSCU < 1). The most frequently used codons are TTT (Phe), ATT (lle), GAA (Glu) and GCT (Ala). Reasons for the bias synonymous codon usage probably arise from different processes (e.g., distinct levels of gene expression, the base composition of genes, gene length and tRNA richness).

According to the RSCU values, codons are classified into optimal codons (RSCU > 1) and non-optimal codon (RSCU < 1). From Fig. 2 and SI Table 3, each amino acid has its preferred codon, with exception of amino acids Met (ATG) and Trp (TGG) that have only one codon and no preference.

A universal genetic code is used for all mitochondrial genes in angiosperms, and the third codon tends to be A or T^58^. A typical translation initiation codon is ATG, but alternative initiation codons occur in translation of *rpl*16^59^, *mtt*B^52^, and *mat*B genes. The initiation codon of the protein-coding genes in the mitochondrial genome of *N. cadamba* is ATG, except for *rps*10 and *rpl1*6 where ACG is the initiation codon.

### Transfer RNA and ribosomal RNA genes

There are 5 tRNA genes in genome 1, with a total length of 371 bp (Table 1). The five tRNA genes range from 71 (tRNA-Arg) to 84 bp (tRNA-Tyr) in length, of which four genes are on the N-strand and one gene is on the J-strand. There are 26 tRNA genes in genome 2, with a total length of 1,909 bp (Table 1). These genes range from 60 bp (tRNA-Val) to 88 bp (tRNA-Ser) in length, of which ten genes are on the N-strand and sixteen genes are on the J-strand.

The secondary structure map of tRNA was predicted and generated using tRNAscan-SE 2.0 (http://lowelab.ucsc.edu/tRNAscan-SE/) ^60^ and ARWEN (Version1.2, http://mbio-serv2.mbioekol.lu.se/ARWEN/) ^61^. Structurally, tRNA-Ser (GCT), tRNA-Ser (TGA) and tRNA-Tyr (GTA) have a group of stem-loop structure on the extra loop between the TψC loop and the anti-codon stem, but the remaining tRNA genes are the typical clover-type secondary structure (SI Fig. 1).

In the secondary structure of tRNA, besides three classic base matches (A-T, G-C and G-T), there are also mismatches, such as G-A, A-C, T-T, T-C and A-A. The T-C and A-A mismatch pairs are only in the anti-codon stems. Three G-A pairs are in the amino acid acceptor stems, and the other three G-A mismatch pairs are in the TψC stems. Two A-C pairs are in the TψC stems, and the other five A-C pairs are in the amino acid acceptor stems. One T-T pair is in the amino acid acceptor stems, and the other four T-T pairs are in the anti-codon stems.

The mitochondrial genome of *N. cadamba* has 3 rRNA genes in total (*rrn*18, *rrn*5, and *rrn*26), ranging from 116 bp to 3,429 bp in length, and all rRNA genes are on the N-strand.

### Repetitive sequences

SI Table 2 indicates that both genome 1 (~ 0.08%) and genome 2(~ 0.16%) have small proportions of repetitive sequences, with the repeat length of 579 bp in total. Most repetitive sequences (microsatellites) consist of single- and di-nucleotide repeats, with more numbers of (A)_n_ and (T)_n_ (14) than (G)_n_ and (C)_n_ (2), and more numbers of (AT)_n_ and (TA)_n_ (8) than others (2 (GA)_n_). Three minisatellites are present in genome 2. All these repeats are not located in protein-coding regions except (T)_10_ in *orf*309 of genome 1 and (A)_10_ in *Atp*1 of genome 2. The small proportion of repeats implies that repetitive sequences do not play an important role in contributing to mitochondrial genome size of *N. cadamba*, different from the patterns of *Nymphaea colorata* ^42^ and other plants ^40^. However, these repetitive sequences could be used to develop molecular markers for population genetic structure analysis in the future.

### Phylogenetic analyses

To assess the taxonomic position of *N. cadamba*, we analyzed the phylogenetic trees species based on complete mitochondrial genomes. Twenty-three species of the asterids-lamiids classification with complete mitochondrial genomes were selected, and *Helianthus annuus* of the non-lamiids classification of the asterids was selected as outgroup. This selection of 22 species of Astragalus was based on tandem sequences of 24 protein-coding genes. The 24 protein-coding genes were 3 adenosine triphosphate synthase genes (*atp*1, *atp*6, *atp*9), 3 cytochrome c oxidase genes (*cox*1, *cox*2, *cox*3), and 1 cytochrome b protein gene (*cyt*B), 9 nicotinamide adenine dinucleotide (NADH) dehydrogenase protein genes (*nad*1, *nad*2, *nad*3, *nad*4, *nad*4L, *nad*5, *nad*6, *nad*7, *nad*9), 4 ribosomal proteins genes (*rps*12, *rps*13, *rps*3, *rps*4) and 4 other genes (*ccm*b, *ccm*c, *ccm*fc, *ccm*fn).

**Figure 3:**
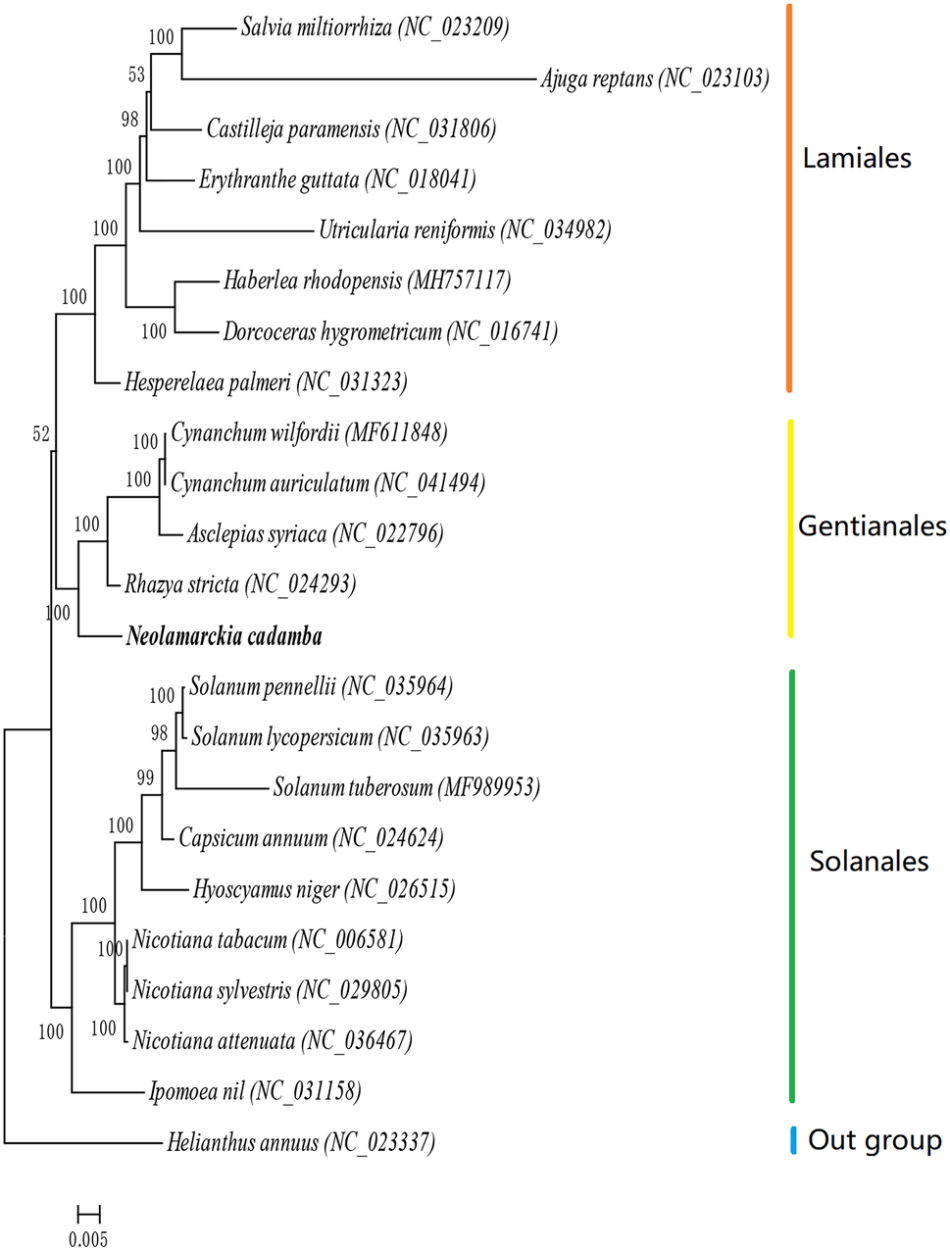
Maximum likelihood tree based on the sequences of 24 PCGs from the mitochondrial genomes of 23 species. The values on branch nodes represent the supporting rates (percentages) derived from 1000 bootstrapping analyses.

JModelTest2.1.7 was used to test the nucleic acid model of the selected sequence DNA^62^, and the best model was GTR + I + G. Maximum likelihood phylogenetic tree was constructed with RAxML8.1.5 software^63^. The clade with *N. cadamba* in Gentianales has two families (Fig. 3): Rubiaceae and Apocynacceae. *Rhazya stricta*, *Asclepias syriaca*, *Cynanchum auriculatum* and *C. wilfordii* in the neighbor branches belong to Apocynacceae, and have closer genetic relationships. *N. cadamba* as the species in family Rubiaceae was earlier differentiated from Apocynacceae. This phylogenetic relationship among the 22 species is consistent with taxonomic groups based on morphological studies.

The *rps*3 gene sequence of 60 species of Rubiaceae was available from NCBI GenBank. Phylogenetic genetic relationships based on this single gene was constructed using the maximum likelihood method. SI Fig. 2 shows that *N. cadamba* is genetically close to *Cephalanthus occidentalis* and *Hymenodictyon parvifolium*. These three species together with *Cubanola domingensis*, *Hillia triflora* and *Rondeletia odorata* provide evidence that they belong to the Cinchonoideae subfamily although *Deppea grandiflora* (Ixoroideae subfamily) and *Guettarda scabra* (Rubioideae subfamily) were incompletely sorted in this clade. Using cpDNA segments (*rbc*L, *rsp*16 intron, *nadh*F, *atp*B–*rbc*L spacer) and nuclear ribosomal ITS, Rydin et al.^64^ showed that five species (*C. occidentalis*, *H. parvifolium*, *C. domingensis*, *H. triflora* and *R. odorata*) belong to Cinchonoideae subfamily. The whole phylogenetic relationships indicate that large genetic divergence and incomplete linage sorting occurred among the three subfamilies of Rubiaceae in terms of the *rps*3 gene sequence.

## Conclusions

In this study, we sequenced the mitochondrial genome of *N. cadamba* and successfully assembled the genome in two maps of circular molecule structure. Genome 1 has 109,836 bp and contains 14 genes. Genome 2 has 305,144 bp and contains 69 genes. The whole genome has slightly high AT content (54.82%). Genome 1 shows negative AT- and GC-skews, while genome 2 shows a negative AT-skew but a positive GC-skew. All protein-coding genes are initiated by the start codon ATG, except for a few genes initiated by alternative codons. The termination codes are TAA for most genes but TGA or TAG for a few genes. Each amino acid has its preferred codon except amino acids Met (ATG) and Trp (TGG) that have only one codon and no preference. The tRNA genes exhibit a typical clover-type secondary structure except tRNA-Ser (GCT), tRNA-Ser (TGA) and tRNA-Tyr (GTA) that have an extra loop between the TψC loop and the anti-codon stem. Tandem repeat sequences are minor, accounting for ~ 0.14% of the whole genome. Phylogenetic analysis with the DNA sequences of 24 PCGs confirms that *N. cadamba* belongs to order Gentianales. Analysis with a single gene *rps*3 of 60 species shows that *N. cadamba* is genetically closer to *Cephalanthus accidentalis* and *Hymenodictyon parvifolium* and belongs to the Cinchonoideae subfamily.

## Methods

### Sample collection and DNA extraction

**Figure 4:**
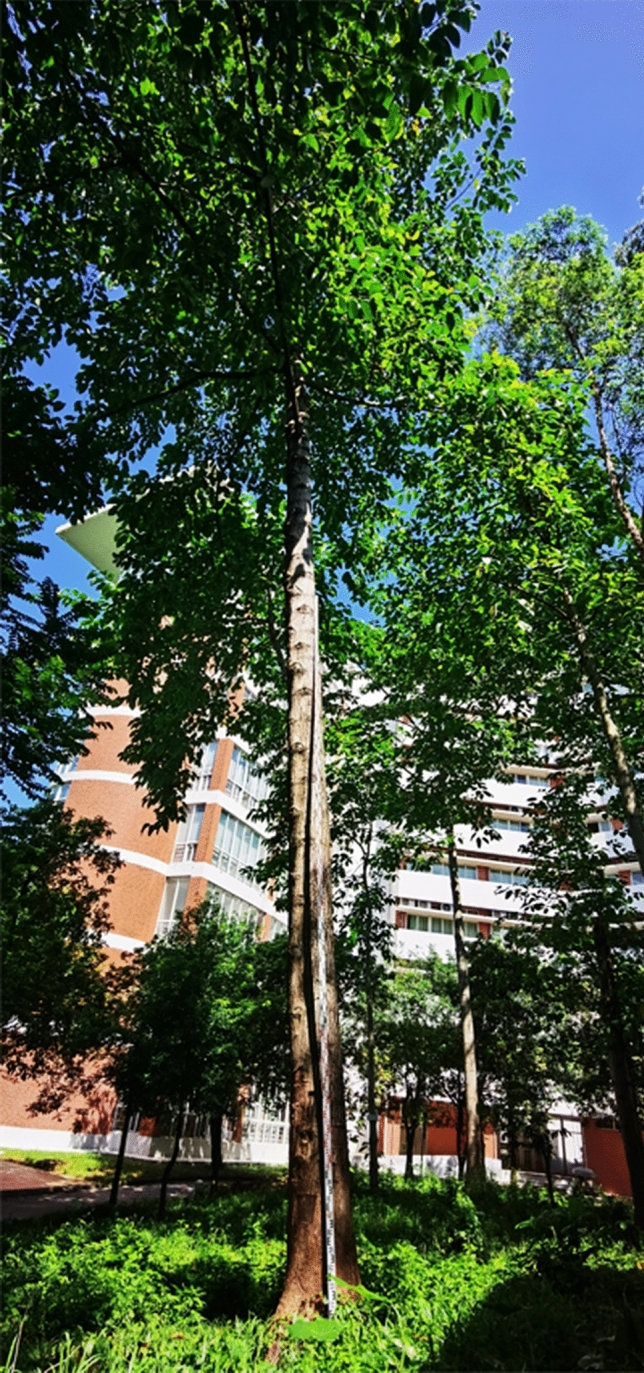
The tree of *Neolamarckia cadamba* from which young leaves were sampled for mtDNA sequencing. The tree grows on campus of South China Agricultural University (23°16′N 113°35′E), Guangzhou, China. It is about 14.5 m in height and 49.04 cm in diameter at the breast height in eleven years.

The leaf sample used in this study was collected from a wild tree (Specimen ID: SCAUNC20190110) on January 10th, 2019. This tree grows on University Campus (23°16′N 113°35′E), South China Agricultural University (SCAU), Guangzhou, Guangdong Province, China. Figure 4 shows the sample tree growing in the fertile and humid soil. XW and XSH identified the voucher specimen and collected leaf samples. The specimen was stored for records in Guangdong Key Laboratory for Innovative Development and Utilization of Forest Plant Germplasm, SCAU, Guangdong Province, China. The use of plant leaves in this study complies with institutional guidelines. Collection of the plant specimen was permitted by the University. Total genomic DNA was extracted from fresh leaves using CTAB method^65^. Then the quality of the extracted DNA samples was tested using (1) 0.8% agarose electrophoresis to detect DNA samples for degradation and impurities, and to estimate the DNA concentration; (2) Nanodrop spectrophotometer to detect the concentration and purity of samples; and (3) Qubit 2.0 Flurometer (Life Technologies, USA) to detect the concentration of samples.

### Library construction and high-throughput sequencing

High-quality genomic DNA of 50 μg was used to generate a 40-kb SMRTbell library, with the size selection on the BluePippin (Sage Science, USA). The genomic DNA library was sequenced on the PacBio sequel platform (Pacific Biosciences, USA). SMRTbell DNA library preparation and sequencing were performed in accordance with the manufacturer’s protocols (Pacific Biosciences, USA), and totally 2 Gb subreads were generated. In order to check the correction of PacBio assembly, an insert size of 500 bp pair-end genomic DNA library for Illumina Hiseq 4000 (Illumina, USA), was constructed by Science Corporation of Gene according to the standard protocol of Illumina. DNA library was constructed after quality control with Agilent 2100 Bioanalyzer (Agilent Technologies, USA). Four gigabytes DNA data were sequenced by Illumina Hiseq 4000 (Illumina, USA).

Different sequencing methods were used in this study because lengths of PacBio sequencing reads were up to 40 kb, which was more suitable for complex genome assembly. However, the PacBio long reads potentially had much more sequencing errors, and the Illumina short reads were then used to fix the errors.

### Genome assembly and annotations

The mitochondrial genome sequence was assembled using Canu (version 2.1, https://github.com/marbl/canu) ^66^ with default parameters on PacBio CLR subreads, and mitochondrial genome sequences were identified with blastn (version 2.10.1 + , https://blast.ncbi.nlm.nih.gov/Blast.cgi) and NCBI nucleotide sequence database. To make improvements of assembly genome with Pilon (version 1.24, https://github.com/broadinstitute/pilon) ^67^, the final PacBio CLR subreads and Illumina clean reads were remapped to mitochondrial genome with bwa (version 0.7.17-r1188, http://bio-bwa.sourceforge.net/) ^68^ and IGV (version 2.9.4, https://igv.org/) ^69^ to confirm. Genome was annotated using DOGMA (http://dogma.ccbb.utexas.edu/) ^70^ and ORF Finder (https://www.ncbi.nlm.nih.gov/orffinder/). For the preliminary results of the annotations, the methods of Blastn and Blastp were used to compare the encoded proteins and rRNA of the reported mitochondrial genome of related species, verify the accuracy of the results and modify them. TRNA was annotated by tRNAscan-SE 2.0 (http://lowelab.ucsc.edu/tRNAscan-SE/) ^71^ and ARWEN (Version1.2, http://mbio-serv2.mbioekol.lu.se/ARWEN/) ^61^, leaving out the tRNA with unreasonable length and incomplete structure, and generating the tRNA secondary structure diagram. Microsatellite identification tool (MISA v2.1) ^72^ and tandem repeat finder (TRF) ^73^ were used to search for repetitive sequences.

### Comparative analysis of mitochondrial genomes

The use of mitochondrial codons had a preference, which would affect the expression of genes and reflect the evolutionary relationship of species to a certain extent. The calculation of relative synonymous codon usage was analyzed with a reference to the formula mentioned in Sharp and Li^74^. The relative synonymous codon usage (RSCU) was calculated as the ratio of the frequency of a focal codon to the mean frequency of all synonymous codons in a given protein-coding sequence. The usage bias of one synonymous codon is indicated when RSCU is not equal to 1; no usage bias is present when RSCU is equal to 1.

In most bacterial genomes, mitochondrial and plastid genomes, there are significant differences in base composition between heavy and light chains, which are called AT-skew and GC-skew. Calculations of the AT- and GC-skews are as follows^75^:

$${\text{AT-skew}} = \frac{A\% - T\% }{{A\% + T\% }},$$

$${\text{GC-skew}} = \frac{G\% - C\% }{{G\% + C\% }}$$

where A%, T%, G% and C% represent the percentages of A, T, G and C in a given sequence, respectively.

### Phylogenetic analyses

MUSCLE v.3.8.31 (http://www.drive5.com/muscle/) software^76^ was used to compare individual genes among multiple species, and then the genes of each species were aligned in a certain order. The protein-encoding gene sequence set of each species was generated by catenating 24 PCG sequences in the same gene order for further analysis. jModelTest2.1.7 (https://code.google.com/p/jmodeltest2/) was used to test the nucleic acid model of the selected sequence DNA^62^, and the best model has the minimum AIC (Akaike Information Criterion) value. Phylogeny tree was constructed with RAxML8.1.5 software (https://sco.h-its.org/exelixis/web/software/raxml/index.html)^63^ using the maximum likelihood (ML) method for both the catenated sequences of 23 species and the *rps*3 gene sequences of 60 species. The bootstrap value was set to be 1000 for each phylogenetic tree analysis.

## Supplementary Information

Supplementary Information.

## Data Availability

mtDNA sequences of *Neolamarckia cadamba* in NCBI GenBank: Genome 1:https://www.ncbi.nlm.nih.gov/nuccore/MT320890.1. Genome 2: https://www.ncbi.nlm.nih.gov/nuccore/MT364442.1.
