# Supplementary material for: A complete sequence of mitochondrial genome of Neolamarckia cadamba and its use for systematic analysis
Source: Sci Rep. 2021 Nov 2;11:21452. doi: 10.1038/s41598-021-01040-9 (PMC8564537; doi:10.1038/s41598-021-01040-9)

A complete sequence of mitochondrial genome of *Neolamarckia cadamba* and its use for systematic analysis

Xi Wang[1,2], Ling-Ling Li[1,2], Yu Xiao[1,2], Xiao-Yang Chen[1,2], Jie-Hu Chen[3], Xin-Sheng Hu[1,2]*

1. College of Forestry and Landscape Architecture, South China Agricultural University, Guangdong 510642, China

2. Guangdong Key Laboratory for Innovative Development and Utilization of Forest Plant Germplasm, South China Agricultural University, Guangdong 510642, China

3. Science Corporation of Gene (SCGene), Guangzhou 510000, China.

*Corresponding author: xinsheng@scau.edu.cn

Supplementary Information

SI Table 1. Composition and skewness of mitochondrial genomes of *Neolamarckia cadamba*

	Feature	Length (bp)	A%	T%	C%	G%	A+T	AT skew	GC skew
Mitochondrial genome 1	Whole genome	109836	26.88	27.57	23.32	22.23	54.45	-0.0128	-0.0241
	PCGs	9663	26.27	28.50	23.75	21.48	54.77	-0.0408	-0.0501
	tRNAs	371	26.15	23.99	27.76	22.10	50.13	0.0430	-0.1135
Mitochondrial genome 2	Whole genome	305144	27.39	27.55	22.38	22.64	54.94	-0.0029	0.0058
	PCGs	27858	27.44	30.75	21.15	20.67	58.18	-0.0569	-0.0115
	tRNAs	1909	24.73	26.24	23.42	25.62	50.97	-0.0298	0.0449
	rRNAs	5386	22.19	26.38	28.91	22.52	48.57	-0.0864	-0.1242

SI Table 2. Repetitive sequences in mitDNA of *Neolamarckia cadamba*

MtDNA genome	SSR	Repeat motifs	Size	Start	End	Rate(%)
1	1	$(T)_{10}$	10	7055	7064	
1	2	$(T)_{10}$	10	29917	29926	
1	3	$(GA)_{6}$	12	35554	35565	
1	4	$(AT)_{6}$	12	48442	48453	
1	5	$(A)_{11}$	11	67085	67095	
1	6	$(A)_{10}$	10	75377	75386	
1	7	$(T)_{10}$	10	85166	85175	
1	8	$(AT)_{7}$	14	87053	87066	
Subtotal			89			0.08
2	1	$(A)_{11}$	11	18797	18807	
2	2	$(C)_{11}$	11	27849	27859	
2	3	$(AT)_{6}$	6	36373	36378	
2	4	$(A)_{10}$	10	36423	36432	
2	5	$(AT)_{8}$	16	74579	74594	
2	6	$(A)_{10}$	10	82485	82494	
2	7	$(T)_{10}$	10	82579	82588	
2	8	$(T)_{11}$	11	89721	89731	
2	9	$(TA)_{6}$	12	101311	101322	
2	10	$(A)_{10}$	10	108973	108982	
2	11	$(AT)_{7}$	14	109872	109885	
2	12	$(G)_{11}$	11	126129	126139	
2	13	$(T)_{11}$	11	135181	135191	
2	14	$(GA)_{7}$	14	158893	158906	
2	15	$(AT)_{7}$	14	179213	179226	
2	16	$(T)_{10}$	10	180116	180125	
2	17	$(TA)_{6}$	12	187777	187788	
2	18	$(T)_{14}$	14	204401	204414	
2	19	$(A)_{10}$	10	211968	211977	
2	20	$(A)_{10}$	10	219481	219490	
2	21	$(ATATAATATAAAAAAAAAG)_{2.1}$	39	37888	37850	
2	22	$(AGAGAAGAAAACGCCTCTCTA)_{2.2}$	45	57669	57714	
2	23	$(GGGGTTGTCGTCTTCAGA)_{2.8}$	51	187642	187692	
Subtotal			490			0.16

SI Table 3. Codon usage of the protein-coding genes in mitochondrial genome of *Neolamarckia cadamba*

Amino acid	Codon	Number	Frequency (%)	RSCU	Amino acid	Codon	Number	Frequency (%)	RSCU
Ala	GCT	345	2.76	1.66	Lys	AAA	324	2.59	1.21
	GCA	200	1.60	0.96		AAG	213	1.70	0.79
	GCC	186	1.49	0.89	Met	ATG	330	2.64	1.00
	GCG	101	0.81	0.49	Phe	TTT	456	3.65	1.16
Arg	CGA	192	1.54	1.41		TTC	330	2.64	0.84
	AGA	191	1.53	1.41	Pro	CCT	253	2.02	1.46
	CGT	167	1.34	1.23		CCA	196	1.57	1.13
	AGG	95	0.76	0.70		CCC	126	1.01	0.73
	CGG	89	0.71	0.66		CCG	119	0.95	0.69
	CGC	81	0.65	0.60	Ser	TCT	272	2.17	1.44
Asn	AAT	263	2.10	1.35		TCA	223	1.78	1.18
	AAC	127	1.02	0.65		AGT	203	1.62	1.07
Asp	GAT	279	2.23	1.38		TCC	172	1.38	0.91
	GAC	124	0.99	0.62		TCG	150	1.20	0.79
Cys	TGT	111	0.89	1.28		AGC	117	0.94	0.62
	TGC	62	0.50	0.72	Stp	TAA	26	0.21	1.77
Gln	CAA	272	2.17	1.53		TGA	12	0.10	0.82
	CAG	84	0.67	0.47		TAG	6	0.05	0.41
Glu	GAA	383	3.06	1.40	Thr	ACT	213	1.70	1.32
	GAG	166	1.33	0.60		ACC	169	1.35	1.05
Gly	GGA	319	2.55	1.50		ACA	163	1.30	1.01
	GGT	275	2.20	1.30		ACG	99	0.79	0.61
	GGG	149	1.19	0.70	Trp	TGG	191	1.53	1.00
	GGC	106	0.85	0.50	Tyr	TAT	298	2.38	1.55
His	CAT	226	1.81	1.51		TAC	86	0.69	0.45
	CAC	74	0.59	0.49	Val	GTA	240	1.92	1.22
Ile	ATT	450	3.60	1.38		GTT	238	1.90	1.21
	ATA	268	2.14	0.82		GTG	168	1.34	0.85
	ATC	260	2.08	0.80		GTC	144	1.15	0.73
Leu	TTA	335	2.68	1.52					
	TTG	275	2.20	1.25					
	CTT	259	2.07	1.17					
	CTA	198	1.58	0.90					
	CTC	140	1.12	0.63					
	CTG	118	0.94	0.53	Total		12507		

SI Figure 1. Secondary structure maps of tRNAs

tRNA-Ala(TGC)

```
          t
        a-t
        g-c
        t+g
        t-a
        t-a
        t+g
        g-c
        a-t     c
 tt    a   tact
t  cagt    !!!! t
c  !!!!    atga g
 t gtca        a
  a   g    t
      a   a
       t+g
       c-g
       g-c
       t.t
       t.t
      c   t
      c   g
       tgc
```

tRNA-Arg(TCG)

```
          c
        g.a
        t+g
        t c
        a-t
        t-a
        g-c
        t+g   ctcaa
       t  tccg    c
 t    a   !!!!    c
c catg    aggc    a
a !!!!     t  ttta
a gtac    a
 g    t   a
       c-g
       t-a
       c-g
       t.t
       t-a
      c   g
      t   a
       tcg
```

tRNA-Asn(GTT)

```
           g
         t-a
         c-g
         c-g
         t+g
         c-g
         a-t
         g+t    ta
        t   catcc  a
 ga    a    !!!!!  a
t  ctcg     gtagg  c
g  !!!!    c    tt
g  gagc     t
 ta    g     g
        g-ctg
        t-a
        c-g
        g+t
        g-c
       c   a
       t   a
        gtt
```

tRNA-Asp(GTC)

```
          g
        g-c
        g-c
        g-c
        a-t
        t+g
        t-a
        g-c    cg
       t   tgccc  a
 taa   a   +!!!!  g
c  cttg    gcggg  c
g  !!+!   t    tt
g  gagc    c
 tca   a   g
       c-gaa
       c-g
       g-c
       c-g
       c-g
       c  a
       t  a
       gtc
```

tRNA-Cys(GCA)

```
            c
           t
         g-c
         g-c
         c-g
         t+g
         a-t
         g+t
         g-c    cc
        t   ctgcc  a
 aa    a    !!!!!  g
t  taca     gacgg  c
g  !!!!     t    tt
g  atgt     a
 aa    a    a
        t+gg
        c t
        g-c
        g-c
        a-t
       c   a
       t   a
        gca
```

tRNA-Gln(TTG)

```
           g
         t-a
         g-c
         g-c
         a-t
         g-c
         t-a
         a-t    ta
        t   tttcc  a
 gaa   a    !!!!!  g
t   ccg     aaagg  c
g   !!!    c    tt
g   ggc     g
 taa   a     t
        t+gca
        c-g
        g-c
        g-c
        t-a
       t   t
       t   g
        ttg
```

tRNA-Glu(TTC)

```
            a
          g+t
          t-a
          c-g
          c-g
          c-g
          t-a
          t-a     tt
         t   tgccc  a
  ga    c    !!!!!  g
 t  cctg     acggg  c
g   !!!!     c    tt
g   ggac     a
 tta    a    g
         t-aa
         c-g
         g-c
         t.t
         c-g
        t   t
        t   a
         ttc
```

tRNA-Gly(GCC)

```
             c
            c
           t
         g-c
         c-g
         g-c
         g-c
         a-t
         a-t
         a c     tg
        t   ctccc  a
 aa    a    !!!!!  a
t  ttcg     gaggg  c
g  +!!!    t     tt
g  gagc     t
 ta    a     g
        t+gag
        a-t
        g-c
        c-g
        c-g
       t   a
       t   a
        gcc
```

tRNA-His(GTG)

```
             c
            c
          g-c
          c-g
          g-c
          g+t
          a-t
          t+g
          g-c     ct
         t   tgccc  a
 tgaa   a    +!!!!  a
g    ccg     gcggg  c
g    !!!    c     tt
a    ggc     g
 tcaa   a     c
         g-cca
         t-a
         g-c
         g-c
         a-t
        t   a
        t   a
         gtg
```

tRNA-IIe(AAT)

```
             t
           a-t
           a-t
           c-g
           a c
           g-c
           a-t
           t-a
           c-g     tat
          t   cactc   c
  tg     a    ! !!!   g
 t  agata     gcgag   t
t   !!!!!          ctc
t   tctat     g
 cac     t   c
          a-t
          t+g
          a a
          a-t
          t+g
         t   t
         t   a
          aat
```

tRNA-Leu(TAG)

```
           c
          a
        c-g
        a-t
        c-g
        g+t
        t-a
        c-g
        c-g     c
 gc    g   gagtg
c  cttt    !!+!+ t
t  !!!+    cttat
 c gaag    t    g
  c   a   a
      a   a
       g-c
       a-t
       a-t
       g+t
       g-c
      t   t
      t   a
       tag
```

tRNA-Lys(TTT)

```
            a
          t-a
          g-c
          g-c
          g-c
          t-a
          g+t
          t-a
          a-t     tg
         t   cgtcc  a
  ga    a    !!!!!  a
 t  ctcg     gcagg  c
t   !!!!    c     tt
g   gagc     t
 gta    a     g
         t-atg
         t-a
         g+t
         g-c
         g-c
        c   a
        t   a
         ttt
```

tRNA-Met(CAT)

```
              ca
             c
            a
          g+t
          g-c
          g-c
          c-g
          t-a
          t-a
          a-t     cg
         t   catcc  a
 taa    a    !!!!!  g
t   tttg     gtagg  c
g   !!!!   t      tt
g   aaac     t
 ttg    g     a
         t+gat
         a-t
         c-g
         c-g
         g-c
        c   a
        t   a
         cat
```

tRNA-Phe(GAA)

```
            g
          g-c
          t+g
          t-a
          c a
          a-t
          g-c
          g+t     ta
         t   tcacc  a
 cga    a    !!!!!  g
t   ctcg     agtgg  c
g   !!!!    c     tt
g   gagc     t
 tta    a     g
         a-tgt
         a-t
         g-c
         g-c
         a-t
        c   a
        t   a
         gaa
```

tRNA-Pro(TGG)

```
             c
            a
          c-g
          g+t
          a-t
          g-c
          g-c
          t-a
          g-c     ta
         t   tgtcc  a
ctga    a    !+!!!  g
t   cgcg     atagg  c
g   !!!!    c     tt
g   gcgc     c
 tca    a     g
         t-agg
         c-g
         t-a
         g-c
         t-a
        t   t
        t   g
         tgg
```

tRNA-Ser(GCT)

```
            g
          g-c
          g-c
          a-t
          g-c
          g-c
          t.t
          a-t     ta
         t   taccc  a
 tga    g    !!!!!  g
g   gtcg     atggg  c
g   :+!!      c    tt
c   aggc     a-t
 tta    a   c t-a
         t-a   a-t
         t+g    c-g
         g-c     a-t
         g+t      a-t
         t-a      g   a
        t   a       aaa
        t   a
         gct
```

tRNA-Ser(TGA)

```
             c
            g
          g-c
          g-c
          a-t
          t-a
          g-c
          g-c
          a-t     ta
         t   ctccc  a
 cga    g    !+!!!  g
g   gtct     ggggg  c
g   :!!!      c    tt
t   aaga     g-c
 tga    g   a t-a
         t-a   a-t
         c-g    t-a
         g-c     t-a
         g-c     g   g
         t-a      a  g
        c   a       ta
        t   a
         tga
```

tRNA-Thr(GGT)

```
            t
          t-a
          g+t
          c-g
          t-a
          g-c
          c-g
          t+g
          t-a   cta
         t   ttc   c
 tga    a    !+!   g
t   gtcg     agg   t
g   !+!:        tgcc
a   cgga     c
 tta    a   g
         g-c
         a-t
         g-c
         a a
         c-g
        c   a
        c   t
         ggt
```

tRNA-Trp(CCA)

```
            g
          g+t
          c-g
          g-c
          c-g
          t-a
          c-g
          t-a     ta
         t   catcc  a
 tga    a    !!+!!  a
t   cttg     gtggg  c
c   !!!!    c     tt
g   gaac     t
 gta    g     g
         t+gat
         g-c
         g-c
         g-c
         t-a
        c   a
        t   a
         cca
```

tRNA-Tyr(GTA)

```
             c
            a
          g-c
          g-c
          g-c
          a-t
          g-c
          a-t
          g-c     ta
         t   cgtcc  a
 tga    g    !+!!!  g
g   gccg     gtagg  c
g   : !!     c    tt
t   aagc     a
 caa    g     t
         a-ttg-c
         c-g  a-t
         a-t   a-t
         g-c   g   t
         a-t     tt
        c   a
        t   a
         gta
```

tRNA-Val(CAC)

```
       a
     g-c
     a.g
     t-a
     t-a
     a c
     t-a
     g-c     g
   ag   gatcg
 ca     !!+!! a
t       ctggc a
t      g     a
 ct     g
   g     g
    t-atc
    g-c
    c-g
    a-t
    c-g
   t   a
   t   a
    cac
```

SI Figure 2. Maximum likelihood tree of 60 species of Rubiaceae based on *rps*3 gene sequences

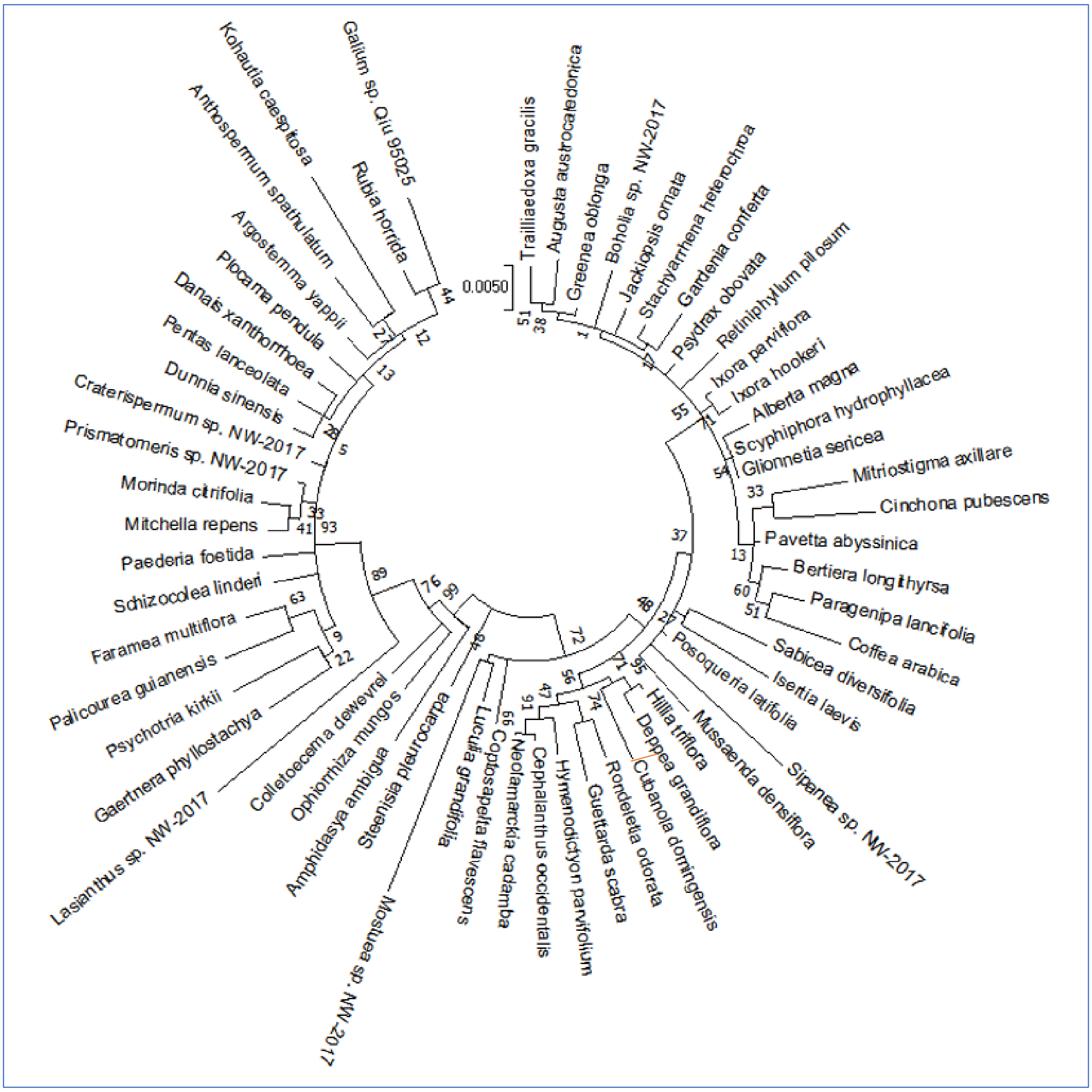

Supplement: Supplementary file 1 — Supplementary Information. [file 41598_2021_1040_MOESM1_ESM.pdf]
